# Simultaneous transcriptional profiling of *Leishmania major* and its murine macrophage host cell reveals insights into host-pathogen interactions

**DOI:** 10.1186/s12864-015-2237-2

**Published:** 2015-12-29

**Authors:** Laura A. L. Dillon, Rahul Suresh, Kwame Okrah, Hector Corrada Bravo, David M. Mosser, Najib M. El-Sayed

**Affiliations:** Department of Cell Biology and Molecular Genetics, University of Maryland, College Park, MD 20742 USA; Center for Bioinformatics and Computational Biology, University of Maryland, College Park, MD 20742 USA; Department of Mathematics, University of Maryland, College Park, MD 20742 USA; Department of Computer Science, University of Maryland, College Park, MD 20742 USA; Present Address: 3128 Bioscience Research Bldg., University of Maryland, College Park, MD 20742 USA

**Keywords:** *Leishmania*, Transcriptome, RNA-seq, Differentiation, Host-pathogen interactions, Macrophage, Mouse

## Abstract

**Background:**

Parasites of the genus *Leishmania* are the causative agents of leishmaniasis, a group of diseases that range in manifestations from skin lesions to fatal visceral disease. The life cycle of *Leishmania* parasites is split between its insect vector and its mammalian host, where it resides primarily inside of macrophages. Once intracellular, *Leishmania* parasites must evade or deactivate the host's innate and adaptive immune responses in order to survive and replicate.

**Results:**

We performed transcriptome profiling using RNA-seq to simultaneously identify global changes in murine macrophage and *L. major* gene expression as the parasite entered and persisted within murine macrophages during the first 72 h of an infection. Differential gene expression, pathway, and gene ontology analyses enabled us to identify modulations in host and parasite responses during an infection. The most substantial and dynamic gene expression responses by both macrophage and parasite were observed during early infection. Murine genes related to both pro- and anti-inflammatory immune responses and glycolysis were substantially upregulated and genes related to lipid metabolism, biogenesis, and Fc gamma receptor-mediated phagocytosis were downregulated. Upregulated parasite genes included those aimed at mitigating the effects of an oxidative response by the host immune system while downregulated genes were related to translation, cell signaling, fatty acid biosynthesis, and flagellum structure.

**Conclusions:**

The gene expression patterns identified in this work yield signatures that characterize multiple developmental stages of *L. major* parasites and the coordinated response of *Leishmania*-infected macrophages in the real-time setting of a dual biological system. This comprehensive dataset offers a clearer and more sensitive picture of the interplay between host and parasite during intracellular infection, providing additional insights into how pathogens are able to evade host defenses and modulate the biological functions of the cell in order to survive in the mammalian environment.

**Electronic supplementary material:**

The online version of this article (doi:10.1186/s12864-015-2237-2) contains supplementary material, which is available to authorized users.

## Background

In order to establish an infection, intracellular pathogens need to adapt to their new environment to survive the innate and adaptive immune responses of the host. A number of human pathogens - including *Leishmania* sp., *Trypanosoma cruzi*, *Mycobacterium tuberculosis*, *Toxoplasma gondii*, *Francisella tularensis*, *Legionella pneumophila*, and *Ehrlichia* - have evolved mechanisms not only to evade the host immune system, but to infect the very immune cells that are recruited to clear an infection [1–7]. *Leishmania* is able to infect and to replicate within mammalian macrophages. It may serve as a model of intracellular infection of immune cells and can be used to study transcriptomic changes that take place in both the host and the pathogen over the course of an infection.

*Leishmania major* and related species are the causative agents of leishmaniasis, a group of diseases that vary in severity from self-healing skin lesions to disfiguring mucosal manifestations to fatal visceral disease. More than a million new cases are reported annually, mostly concentrated in the Middle East and Central and South America [8]. The *Leishmania* life cycle is divided between its insect vector, the phlebotomine sand fly, and its mammalian host, where it resides primarily inside of macrophages although neutrophils, dendritic cells, and fibroblasts have also been implicated at various stages of infection [9–13]. Previous studies have shown that the parasite undergoes changes in morphology and alterations in cell surface components as it adapts to the intracellular environment [14–16]. Additionally, a CD4+ T helper (Th) type 1 response by the host leads to parasite killing while a Th2 response leads to parasite growth [17–19]. Less is known about the global changes that take place at the transcriptomic level in both the parasite and host over the course of an infection.

Once in host macrophages, *Leishmania* parasites rapidly transform into aflagellated amastigote forms, which are contained inside of parasitophorous vacuoles. The parasite enters host cells by receptor-mediated phagocytosis. It is thought to do so in a quiescent manner, failing to produce a significant oxidative burst and to activate the innate immune system [20–23]. *Leishmania* prevent their killing by altering cytokine expression (thereby influencing T cell responses), impeding antigen display by MHC class II molecules, and hindering nitric oxide production (reviewed in [24–26]).

Previous studies using microarrays have started to elucidate changes that occur within the parasite or within the host as infection occurs [27–47], but have so far not sought to look at the transcriptomes of both simultaneously and over the course of an intracellular infection. Studies on host response have identified genes that are differentially regulated upon infection with various *Leishmania* species, sometimes with opposing results. Some of these differences may be attributable to the parasite species and host systems used, the severity of the resulting infection, and the timepoints examined. Additionally, studies of *Leishmania* amastigotes have often used axenic cultures [35, 36, 38, 45, 47] or lesion-derived amastigotes [27, 28, 35, 37, 44, 48]. The former have been shown to significantly differ from the intracellular biological state [35, 45] while the latter contain a mixture of amastigotes at various timepoints post-infection, making it difficult to differentiate between changes that take place early versus later during intracellular infection.

In this study, we performed transcriptome profiling using RNA-seq to concurrently identify global changes in host and parasite gene expression that occur over the first 72 h of a synchronized intracellular infection of murine peritoneal macrophages with *Leishmania major*. A paired-end mRNA sequencing approach was used to allow high-confidence read mapping and transcript assembly. Collection of data from multiple biological replicates, the use of matched host control samples, careful statistical analysis of variation, and removal of batch effects provided us with a unique ability to detect biological differences between samples and timepoints with high confidence and sensitivity. Differential gene expression analysis enabled us to identify host and parasite genes that were modulated over time, and pathway and gene ontology analyses provided insights into the higher level processes activated during infection. This work builds on and improves existing expression datasets and provides insights into how *Leishmania* is able to evade host defenses and cause modulations in the host transcriptome in order to survive in the mammalian intracellular environment.

## Results and discussion

### Infection dynamics and global transcription patterns

**Fig. 1:**
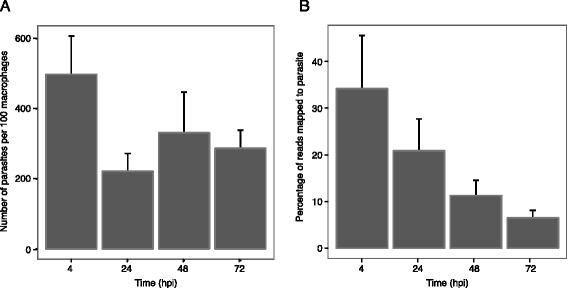
Characterization of *L. major* intracellular growth and proportion of RNA from the parasite. Mouse macrophages infected with *L. major* were collected at 4, 24, 48, and 72 hpi and subjected to transcriptional profiling by RNA-seq. An average of 87 % of macrophages were infected across all samples. Bar plots are used to illustrate **a** the average number of parasites observed per 100 host cells and **b** the average percentage of trimmed RNA-seq reads that map to the *L. major* genome. Standard errors bars are shown. No statistically significant changes were observed between timepoints

Transcriptome profiling by RNA-seq was used to identify global changes in gene expression over the course of the first 72 h of an infection of murine macrophages with *Leishmania major*. Peritoneal macrophages from C57BL/6 mice were infected with *L. major* metacyclic promastigotes and samples collected at 4, 24, 48, and 72 h post-infection (hpi). The dynamics of the infection were monitored by counting the number of parasites per 100 macrophages (Fig. 1a). RNA sequencing was carried out for each sample and for matched uninfected controls, as well as for the *L. major* metacyclic promastigotes used for the infection. Over 2.4 billion sequence reads were collected across three independent experiments (biological replicates) and labeled as batches A-C (see Additional file 1).

Since there is little sequence conservation between mouse and *L. major*, we were able to unambiguously map reads from mouse and parasite RNAs from the mixed sample to their respective genomes. For uninfected samples, the percentage of reads mapping to the mouse genome ranged from 88.9 to 95.9 %. Greater heterogeneity was observed for the percentage of reads mapping to *L. major* in the infected samples, providing clues about the transcriptional activity of both parasite and host (Fig. 1b). For each batch, the percentage of reads mapping to the parasite decreased over the course of the infection (see Additional file 1). Interestingly, this decrease in the relative portion of parasite reads did not match the parasite load of the infected cells, which decreased sharply between 4 and 24 h for each batch and remained constant or slowly rose again from 24 through 72 h (Fig. 1a and Additional file 1). Possible explanations for the mismatch between the constant/increasing number of parasites and the decreasing percentage of parasite reads is that the individual parasites are less transcriptionally active as the infection progresses or that the mouse cells became more transcriptionally active over the course of the infection, thereby diluting the proportion of reads attributable to the parasite.

Samples from host and from parasite showed a similar distribution of per-gene read counts per sample, as visualized by box plots (Additional file 2: Figure S1), but striking differences were observed when comparing the two organisms. The median steady-state expression level was elevated in *L. major* compared to mouse (6.0 vs. 3.6 log2 counts per million) and *L. major* genes showed a much more narrow distribution (interquartile ranges of 5.6 to 6.5 for *L. major* and 0 to 6.8 for mouse). Additionally, when non- and lowly-expressed genes were removed from the datasets prior to differential expression analysis, this filter led to the removal of 10,548 genes from the mouse dataset, but only 7 genes from the parasite one.

The differences in the distribution of genes between mouse and *L. major* are consistent with differences in how each organism controls gene expression - mouse employs both transcriptional and post-transcriptional mechanisms to control the expression levels of individual genes, resulting in many genes that are not expressed and a wide dynamic range for those that are. This differs from *Leishmania*, which employs polycistronic transcription by RNA polymerase II at roughly the same rate across its genome [49, 50]. The resulting polycistronic mRNAs are split into their component mature mRNAs by coupled *trans*-splicing and polyadenylation events [51–53]. Steady-state mRNAs levels of individual genes are thus not determined by the amount of transcription, but by post-transcriptional processes such as RNA processing and degradation (reviewed in [54]). The dearth of lowly expressed *L. major* genes may indicate that very few mRNAs are completely degraded following polycistronic transcription.

### Statistical assessment of biological replicates and batch effects

The use of multiple biological replicates necessitated the evaluation of the data to assess reproducibility and account for batch effects, i.e. experimental variation caused by sub-groups of measurements that are not related to the underlying biology of the system being studied. Previous analyses of high-throughput data, like those produced by RNA-seq, have indicated the need to assess and correct batch effects in order to prevent misinterpretation of results [55, 56]. In this study, experimental start date was used as a surrogate for batch when testing for differential expression.

**Fig. 2:**
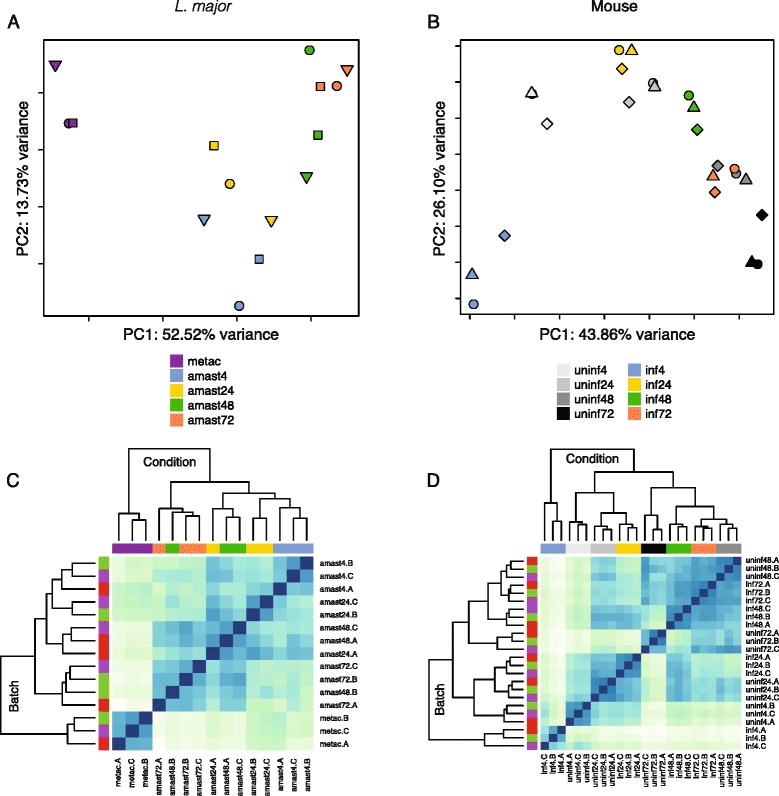
Global gene expression profiles of *L. major* parasites and their murine macrophage host cells. RNA-seq was carried out on mouse macrophages infected with *L. major* at 4, 24, 48, and 72 hpi as well as on the metacyclic promastigotes used for the infection. Principal component analysis (PCA) plots and heatmaps of hierarchical clustering analyses using Euclidean distance are shown for the *L. major* (**a, c**) and mouse (**b, d**) transcriptomes over the course of the experiment. The analyses were performed using all annotated protein-coding genes following filtering for low counts and quantile normalization after accounting for batch effects in the statistical model (8479 genes for *L. major* and 12552 genes for mouse). In the PCA plots, the first two principal components are shown on the X and Y axes, respectively, with the proportion of total variance attributable to that PC indicated. Each experimental sample is represented as a single point with color indicating sample type/timepoint and shape indicating experimental batch. Colors along the tops of the heatmaps indicate the sample type/timepoint and colors along the left sides of the heatmaps indicate the experimental batch. Samples are named according to sample type (“metac” for *L. major* metacyclic promastigotes, “amast” for *L. major* amastigotes, “uninf” for uninfected mouse macrophage, or “inf” for *L. major*-infected mouse macrophage), timepoint (4, 24, 48, or 72 hpi) and experimental batch (**a, b**, or **c**) (see Additional file 1)

We used principal component analysis (PCA) and Euclidean distance heatmap analysis to visualize the relationship between experimental datasets both prior to and after adjusting for batch effects (Fig. 2). The PCA plots revealed that samples from the same experimental condition (hpi and infection status) grouped together for the parasite and mouse macrophages demonstrating a high level of reproducibility between biological replicates. The dendrograms associated with the Euclidean distance heatmaps further illustrated this point, with like samples clustering most closely with one another. The grouping of samples by experimental condition was partially obscured when batch effects were not considered, illustrating the benefits of this approach (Additional file 2: Figure S2). Batch effects were therefore controlled for in the subsequent differential expression analyses by including experimental batch in the statistical models used.

The PCA and clustering analysis also suggested interesting biological relationships between the samples. The global profile of *L. major* gene expression changed over the time course of the experiment, moving from left to right across principal component (PC) 1 (Fig. 2a). The clear separation between promastigote and amastigote samples along PC1 highlights the differences between the two developmental stages. While a time progression can be seen within the intracellular amastigote samples, some intermixing of samples from different timepoints was observed beyond 4 hpi (Fig. 2c), suggesting an overlap in the gene expression profiles for these samples.

A time progression was also seen in the mouse macrophage data for both infected and uninfected samples (Fig. 2b and d). This observation underscored the necessity of collecting uninfected controls from each timepoint studied rather than relying on a control from a single timepoint. In addition, all infected macrophages from 24 to 72 hpi clustered more closely with the uninfected macrophages while the 4 hpi infected macrophages clustered apart. This is especially apparent in the heatmap dendrogram where the 4 hpi infected macrophages appear as a major outgroup.

### Identification of differentially expressed genes and pathway-based enrichment analyses in murine host macrophages

While clustering analyses provided a high level overview of the behavior of the murine macrophage and parasite transcriptomes during the infection, further analyses were needed to evaluate changes in the expression levels of individual genes. A differential expression analysis was carried out to closely dissect the host murine macrophage response to infection by *Leishmania major* at 4, 24, 48, and 72 h after infection. Pairwise analyses were conducted within and across individual timepoints with infected macrophages evaluated against matched uninfected macrophages for each timepoint.

**Fig. 3:**
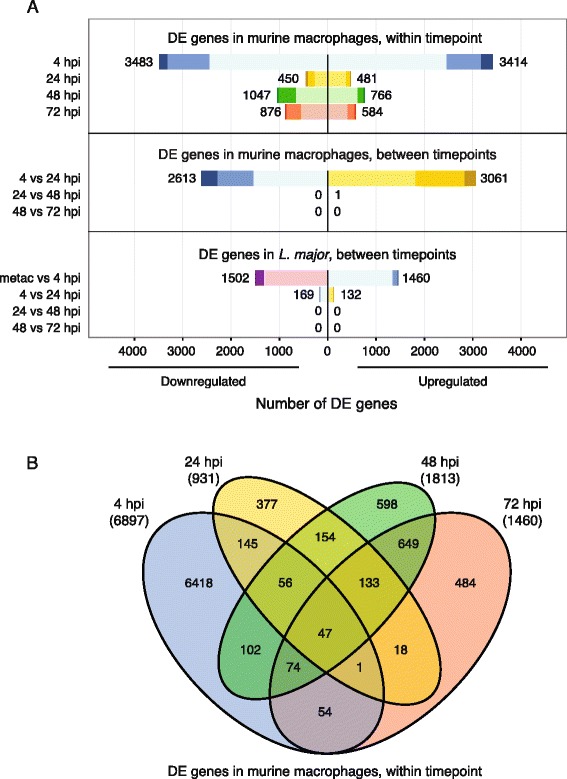
Differentially expressed genes in *L. major* parasites and their murine macrophage host cells. RNA-seq was carried out on mouse macrophages infected with *L. major* at 4, 24, 48, and 72 hpi as well as on the metacyclic promastigotes used for the infection. Pairwise comparisons were done to determine differentially expressed (DE) genes from uninfected vs. infected mouse samples at each timepoint (**a**, *top*) and between timepoints (**a**, *middle*), and for *L. major* parasite samples between timepoints (**a**, *bottom*). Box length depicts the number of DE genes either downregulated (left) or upregulated (right) at an adjusted *P* value of < 0.05 with the total number of down- and upregulated genes shown. Color hue indicates sample type/timepoint as defined in Fig. 2 and color shade indicates the proportion of genes with > 4-fold differential expression (*dark*), between 2- and 4-fold differential expression (*medium*), or 2-fold differential expression (*light*). The DE gene lists for uninfected vs. infected mouse cells at each timepoint were compared and the overlap shown as a Venn diagram in (**b**). The complete lists of DE genes are provided in Additional file 3 for mouse and Additional file 6 for *L. major*

The most substantial response to infection by the macrophage was observed at 4 hpi, with 6897 genes differentially expressed (DE) between uninfected and infected cells with fold changes (FC) ranging from 29-fold downregulated to 56-fold upregulated. The response is reduced in later timepoints as reflected in smaller numbers of DE genes - 931, 1813, and 1460 genes at 24, 48, and 72 hpi, respectively - and in reduced fold changes with no downregulation beyond 12-fold or upregulation beyond 18-fold for these timepoints. The breakdown of up- and downregulated genes for each timepoint comparison is illustrated in Fig. 3a (top) and Additional file 2: Figure S3**.** Complete DE gene lists for each timepoint are provided in Additional file 3.

A direct comparison of the overlap in DE genes at each timepoint revealed that the macrophage response to infection at 4 hpi was vastly different from the response at the later timepoints (Fig. 3b and Additional file 2: Figure S4). Of the 6897 DE genes at 4 hpi, 93 % (6418 genes) were unique to 4 hpi. This is in contrast to 40 % of genes at 24 hpi (377), 33 % of genes at 48 hpi (598), and 33 % of genes at 72 hpi (484) that were DE only at that specific timepoint. Indeed, only 47 genes were consistently up- or downregulated at all 4 timepoints (38 genes upregulated at all timepoints and 9 genes downregulated at all timepoints; see Additional file 3). These genes do not appear to be functionally related, although the list does include the heavy metal transporters metallothionein 1 and 2. The latter has previously been associated with *Leishmania* resistance to treatment with antimonial drugs [57]. Interestingly, two genes associated with *Bcl2* (*Bnip3* and *Bcl2a1b*), an inhibitor of apoptosis, are also on this list, suggesting that infection by *L. major* may induce changes in host gene expression to prevent macrophages from undergoing cell death.

In order to detect statistically significant differences in gene expression over time, we conducted differential expression analysis across timepoints. Contrasts between successive timepoints revealed a large number of DE genes during the 4 to 24 hpi transition (5674 DE genes), but almost no statistically significant genes between 24 and 48 hpi (1 gene) or between 48 and 72 hpi (0 genes) (Fig. 3a middle, Additional file 2: Figure S5B, and Additional file 3). This suggests that the large initial response of the murine macrophages to *L. major* infection stabilized by 24 hpi with no additional changes detected for the remainder of the time course as the infected cells mostly maintain their expression profile relative to matched uninfected cells.

The cellular processes most affected at each timepoint were characterized by KEGG pathway enrichment analysis using ConsensusPathDB [58]. Genes that were DE >2-fold were used as input with upregulated and downregulated genes considered separately. The results of this analysis are reported in Table 1, Additional file 2: Table S1 and Table S2, and Additional file 4.

**Table 1:** KEGG pathways enriched in murine macrophages at 4 hpi. KEGG pathway analysis using ConsensusPathDB [58] identified signaling and metabolic pathways that were over-represented in *L. major*-infected mouse macrophages at 4 hpi (*P* value < 0.01) relative to uninfected controls. Genes that were differentially expressed (DE) by more than 2-fold were used as input with up- and downregulated genes considered separately. For each enriched KEGG pathway, the number of DE genes assigned to that pathway, the total number of genes in the pathway, and the *P* value for the enrichment are reported. The differentially expressed genes corresponding to each enriched KEGG pathway are reported in Additional file 4

KEGG pathway, upregulated	Number DE Genes	Pathway Size	*P* Value
Cytokine-cytokine receptor interaction	43	266	3.49e-12
TNF signaling pathway	24	111	3.05e-09
Transcriptional misregulation in cancer	24	180	5.72e-09
HIF-1 signaling pathway	20	113	4.57e-07
Hematopoietic cell lineage	16	87	5.84e-07
NF-kappa B signaling pathway	19	102	1.55e-06
Jak-STAT signaling pathway	22	155	2.24e-06
Legionellosis	13	59	1.05e-05
PI3K-Akt signaling pathway	37	353	6.29e-05
MAPK signaling pathway	28	257	2.57e-04
Salmonella infection	12	79	9.24e-04
Malaria	9	49	1.00e-03
NOD-like receptor signaling pathway	10	59	1.03e-03
Rheumatoid arthritis	12	84	1.60e-03
Glycolysis/Gluconeogenesis	9	65	2.19e-03
Mineral absorption	8	46	2.72e-03
ECM-receptor interaction	11	87	6.42e-03
Tuberculosis	18	177	6.45e-03
Gap junction	11	88	7.00e-03
Axon guidance	14	129	8.88e-03
Arginine and proline metabolism	8	56	9.30e-03
Prion diseases	6	35	9.77e-03
KEGG pathway, downregulated	Number DE Genes	Pathway Size	*P* Value
Osteoclast differentiation	21	126	2.99e-06
Terpenoid backbone biosynthesis	7	21	7.30e-05
Staphylococcus aureus infection	11	52	7.61e-05
Steroid biosynthesis	6	17	1.71e-04
NOD-like receptor signaling pathway	10	59	2.51e-04
Peroxisome	12	81	1.18e-03
PPAR signaling pathway	12	81	1.18e-03
Chagas disease (American trypanosomiasis)	14	104	1.24e-03
Biosynthesis of unsaturated fatty acids	6	25	1.69e-03
Leishmaniasis	10	66	2.50e-03
ABC transporters	8	46	2.75e-03
Axon guidance	15	129	3.69e-03
Leukocyte transendothelial migration	14	121	5.16e-03
Fc gamma R-mediated phagocytosis	11	89	7.70e-03
Pancreatic cancer	9	67	9.02e-03

The enriched KEGG pathways were considered alongside the DE gene lists to gain insights into the cellular response to infection and how it differs between early and later infection. Early in the infection (4 hpi), many of the KEGG pathways that are most highly induced in infected macrophages are related to immune responses, specifically cytokine-cytokine receptor interactions and arginine and proline metabolism, glycolysis, and multiple signaling pathways including those for TNF, HIF-1, NF-kappa B, Jak-STAT, PI3K-Akt, and MAPK. A closer look at the DE genes in the immune response-related pathways reveals an interesting picture of the murine macrophage response to infection at 4 hpi. Infected macrophages produce a set of transcripts with paradoxical functions involved in both activating immune responses and promoting tissue regeneration and wound healing. Many of the DE genes associated with enriched pathways are anti-inflammatory in character or are involved in tissue growth and repair, including *Csf1*, *Csf3*, *Il10*, *Il11r*, *Il1rn*, *Socs3*, *Hmox1*, *Egfr*, *Vegf*, and fos-induced growth factor (*Figf*). The product of each of these genes has either been associated with reducing inflammation or promoting cell survival or differentiation. Therefore, macrophages infected with *L. major* appear to assume an immunoregulatory phenotype that resembles previously described macrophages treated with LPS and immune complex [59]. However, not all of the differentially induced genes in infected macrophages at 4 hpi were anti-inflammatory. There was also an upregulation of transcripts encoding well-described inflammatory cytokines and their receptors including *Il1*, *Il6*, *Tnf*, and *Nos2*, as well as *Il1rap* and *Il18r1*. The pro-inflammatory NOD-like receptor signaling pathway is enriched among both upregulated and downregulated genes at 4 hpi with some members of the pathway upregulated (*Tnf*, *Il6*, *Il1β*, *Cxcl2*) and others downregulated (NLRP-family members, *Pycard*, *Card9*, and *Il18*). It is unclear to what extent the observed expression patterns are being driven by the parasite as it is taken into the host cell, or are being promoted by the host as it attempts to limit infection or to protect itself from the negative effects of a robust immune response.

Pathway analysis also showed an upregulation of glycolysis/gluconeogenesis at 4 hpi. Upregulated genes encode glycolytic enzymes, including hexokinases, enolase, phosphoglycerate kinase, glyceraldehyde-3- phosphate dehydrogenase, and lactate dehydrogenase A. An increase in anaerobic glycolysis has been associated with the stimulation of inflammatory responses in macrophages following TLR ligation [60]. Glycolytic pathways were also enriched among upregulated genes at 24 hpi, but this effect was not seen at 48 and 72 hpi. Thus, following phagocytosis of *L. major*, macrophages undergo transient metabolic alterations that result in an increase in glycolysis and the generation of ATP.

KEGG pathways downregulated at 4 hpi are mostly related to lipid metabolism and biogenesis and include osteoclast differentiation, terpenoid backbone biosynthesis, steroid biosynthesis, PPAR signaling, and biosynthesis of unsaturated fatty acids. Decreases in the receptors and signaling molecules involved in the process of phagocytosis itself (“Fc gamma R-mediated phagocytosis” KEGG pathway) were also observed at 4 hpi. It has been previously shown that phagocytosis of *Leishmania* and other pathogens via Fc gamma receptors leads to parasite killing while phagocytosis via pathways mediated by complement receptor 3 (CR3) leads to parasite survival [61–65]. The downregulation of Fc gamma receptors may be a mechanism by which the parasite induces changes in host cells to promote alternative entry mechanisms that will support its survival. While we observed a slight downregulation of mRNA levels for C3ar1, C5ar1, and C5ar2 early in infection, upregulation of C5ar2 was detected at 48 and 72 hpi.

An examination of the enriched KEGG pathways at 24, 48, and 72 hpi reveals different enrichment patterns from those observed at 4 hpi (Additional file 2: Table S1 and Table S2 and Additional file 4). There are a greater number of pathways downregulated than upregulated during each of these later timepoints, but no clear picture otherwise emerges.

While a significant number of disease-specific KEGG pathways are enriched among up- and downregulated genes reported at various timepoints in this study (i.e., leishmaniasis, Chagas disease, tuberculosis, malaria, legionellosis, amoebiasis, *Salmonella* infection, *Staphylococcus aureus* infection, Fanconi anemia, prion diseases, and cancer subtypes; see Table 1 and Additional file 2: Table S1 and Table S2), a linkage between these disease and our dataset may not be particularly meaningful. This is because the KEGG pathways for these individual diseases appear to be constructed using a combination of heterogeneous observations from multiple studies of individual genes using data from different experiments, hosts, cell types, and timepoints, rather than using gene lists derived from a single or set of genome-wide studies, and as a result paint an incomplete picture of the global changes in gene expression. Therefore, while it is interesting to note small overlaps between the comprehensive DE profiles in this study and genes included in these pathways, the differences in scope between the lists limit the usefulness of comparisons to disease KEGG pathways as currently defined.

We compared the results of our differential expression analysis to previously published reports on the murine macrophage response to *Leishmania* infection. Since global transcriptome studies of *L. major* infection of macrophages isolated from C57BL/6 mice are not available, we compared our results to studies that used either the same mouse strain or the same *Leishmania* species (see Additional file 5). In a study detailing the infection of C57BL/6 peritoneal macrophages with *L. amazonensis* [41], 105 genes were identified as DE (FC > 1.5) between infected and uninfected macrophages at 18 hpi. This number compares to 3972 and 546 DE genes (FC > 1.5) at 4 and 24 hpi, respectively, in our dataset with an overlap of only 42 genes (of the 105) differentially expressed in the same direction at 4 or 24 hpi. Similarly, we compared the infection of Balb/c bone marrow-derived macrophages with *L. major* as reported in two recent studies. Of the 769 genes identified when comparing the transcriptomes of *L. major*-infected Balb/c macrophages to macrophages that had ingested latex beads (24 hpi timepoint), only 53 were in common with our 24 hpi timepoint [34]. However, 446 genes were in common with our 4 hpi timepoint, indicating that the time progression observed between the two systems may be somewhat offset. Of the 40 genes identified as DE by more than 1.5-fold during the 24 h timecourse experiment reported by Rabhi et al. [42], 29 genes were also found to be differentially expressed in the same direction in our dataset at either 4 or 24 hpi with the greatest number of genes found in common with our 4 hpi dataset (26 genes). The limited agreement between the previously reported DE genes and our dataset is likely a reflection of the differences in study design (e.g., combination of mouse strain and parasite species, macrophage source, parasite opsonization), batch effects between laboratories, technical platforms, and data analysis methods.

### Identification of differentially expressed genes and gene ontology-based enrichment analyses in *L. major* parasites

While past studies have used microarrays and other methods to study changes in *Leishmania* gene expression between promastigotes and amastigotes [27, 28, 31, 35, 37, 38, 44, 47, 66, 67], this study provided a unique opportunity to additionally characterize gene expression patterns within amastigotes over the course of an intracellular infection. Differential expression analysis revealed a large number of genes (2962) to be DE between metacyclic promastigotes and 4-h amastigotes at an adjusted *P* value cutoff of <0.05, reflecting the significant differentiation event that occurs as the parasite enters host cells. Significantly fewer changes in expression were observed in amastigotes across timepoints, with 301 DE genes found between 4- and 24-h amastigotes and no DE genes between either 24- and 48-h amastigotes or between 48- and 72-h amastigotes (Fig. 3a, bottom, Additional file 2: Figure S5A, and Additional file 6). This pattern of differential gene expression is consistent with observations of gene expression over time in the mouse macrophages and suggests that the initial reprogramming that occurs upon infection had largely been stabilized by 24 hpi.

Gene ontology (GO) analysis was used to identify cellular functions and processes that characterize the entry and survival of *L. major* in the murine macrophage system. These results were considered alongside the lists of DE genes to draw insights into how the parasite adapts to its new environment. Efforts were made to compare the results of this study to previous work, though our ability to conduct meaningful systematic comparisons was constrained due to the use of different parasite systems (e.g., *Leishmania* species, source of amastigotes, developmental stages and timepoints collected), host systems (e.g., mice strains, cell lines), experimental platforms (e.g., microarrays, number of genes interrogated), and methods for assessing and reporting differential expression (fold change vs. statistical cut-offs; details of reported gene lists).

GO analysis of the up- and downregulated genes identified during the metacyclic promastigote to 4-h amastigote transition revealed a total of 20 enriched GO categories. Enriched GO terms for genes upregulated during this transition were primarily related to mitigating the effects of an oxidative response by the host immune system and the regulation of proteins (Table 2 and Additional file 7). Heat shock proteins, especially HSP83, multiple tryparedoxin peroxidase family members, and multiple cyclophilins were upregulated upon entry of metacyclic promastigotes into host cells and contributed strongly to the GO enrichment results for the upregulated genes. HSP83, the cytoplasmic form of HSP90, is known to stabilize transcription factors and kinases and is thus largely involved in the regulation of signaling networks involved in differentiation [68, 69], and tryparedoxin peroxidase is known to contribute to *Leishmania* virulence and resistance to antimonial drugs [70]. Less is known about the role of cyclophilins in *Leishmania*, though these peptidyl-prolyl cis/trans isomerases have been shown to reactivate cellular proteins by promoting their disaggregation [71]. The upregulation of cyclophilin in *L. major* amastigotes has been previously observed (compared to procyclic promastigotes) [37]. Many types of surface antigens were highly upregulated, as was the zinc-metalloprotease GP63 (a known virulence factor that subverts macrophage signaling) [72], and phosphoglycan beta 1,3 galactosyltransferase (SCG) family members, which are responsible for modifications to the *Leishmania* lipophosphoglycan (LPG) surface molecule side chains [73] and have been previously observed to be upregulated in *Leishmania* amastigotes [44]. That there are significant changes taking place on the surface of the parasite is not surprising given the transformation that takes place as differentiation progresses from the promastigote to amastigote forms. Other genes upregulated during the metacyclic promastigote to 4-h amastigote transition included *meta1,* which is thought to play a role in virulence and was previously found to be upregulated in infective metacyclic promastigotes relative to non-infective procyclic promastigotes [55, 74, 75], and inositol-3-phosphate synthase (*ino1*), which synthesizes *myo*-inositol, a precursor molecule for the backbone of the GPI anchors used by many *Leishmania* surface proteins, including multiple virulence factors [76]. Interestingly, the gene encoding the transporter for *myo*-inositol (*mit1*) [77] was among the most downregulated during the transition, perhaps indicating that the parasite favors synthesizing *de novo* rather than importing this important substrate once it enters mammalian cells.

**Table 2:** Gene ontology (GO) categories enriched across *L. major* stages/timepoints. GOseq [101] was used to identify enriched GO categories for the transition between metacyclic promastigotes and 4-h amastigotes and between 4-h amastigotes and 24-h amastigotes at a *P* value cutoff of < 0.05. For each transition, up- and downregulated genes were considered separately. The category for each enriched GO term is indicated (BP = biological process, MF = molecular function, CC = cellular component). The differentially expressed genes corresponding to each enriched GO category are reported in Additional file 7

GO ID	GO term	*P* value
Metacyclics to 4-h amastigotes, upregulated		
GO:0006950	response to stress (BP)	5.50E-13
GO:0006486	protein glycosylation (BP)	9.95E-09
GO:0006457	protein folding (BP)	5.70E-06
GO:0051920	peroxiredoxin activity (MF)	2.72E-05
GO:0051082	unfolded protein binding (MF)	9.44E-05
GO:0016209	antioxidant activity (MF)	1.04E-04
GO:0004386	helicase activity (MF)	2.06E-04
Metacyclics to 4-h amastigotes, downregulated		
GO:0004713	protein tyrosine kinase activity (MF)	6.68E-12
GO:0004674	protein serine/threonine kinase activity (MF)	2.15E-11
GO:0004672	protein kinase activity (MF)	3.40E-10
GO:0006468	protein phosphorylation (BP)	3.43E-10
GO:0005516	calmodulin binding (MF)	2.76E-08
GO:0009434	microtubule-based agellum (CC)	1.93E-07
GO:0005840	ribosome (CC)	8.85E-07
GO:0006412	translation (BP)	1.42E-06
GO:0003735	structural constituent of ribosome (MF)	1.81E-06
GO:0006633	fatty acid biosynthetic process (BP)	2.72E-05
GO:0009190	cyclic nucleotide biosynthetic process (BP)	8.29E-05
GO:0016849	phosphorus-oxygen lyase activity (MF)	8.29E-05
GO:0007165	signal transduction (BP)	2.66E-04
4-h to 24-h amastigotes, upregulated		
GO:0005874	microtubule (CC)	3.25E-09
GO:0043234	protein complex (CC)	5.80E-09
GO:0051258	protein polymerization (BP)	5.80E-09
GO:0005198	structural molecule activity (MF)	3.32E-08
GO:0006184	GTP catabolic process (BP)	3.91E-07
GO:0007018	microtubule-based movement (BP)	9.88E-06
GO:0003924	GTPase activity (MF)	1.81E-05
4-h to 24-h amastigotes, downregulated		
GO:0055114	oxidation-reduction process (BP)	8.81E-08
GO:0006096	glycolytic process (BP)	1.24E-07
GO:0020015	glycosome (CC)	4.22E-07
GO:0004743	pyruvate kinase activity (MF)	1.50E-05
GO:0050661	NADP binding (MF)	2.36E-05
GO:0004365	GAPDH (NAD+) (phosphorylating) activity (MF)	5.91E-05
GO:0006006	glucose metabolic process (BP)	1.45E-04

GO terms that were enriched among downregulated genes during the metacyclic promastigote to 4-h amastigote transition were related to translation, cell signaling, fatty acid biosynthesis, and flagellum structure (Table 2 and Additional file 7). A number of genes were responsible for driving the GO results, including ribosomal proteins, casein kinase, receptor-type adenylate cyclase, fatty acid elongase, sphingolipid delta desaturase, and paraflagellar rod protein. The downregulation of ribosomal proteins during the *Leishmania* promastigote to amastigote transition is consistent with previous reports [28] and may reflect a reduction in translation taking place within the cell. Casein kinase is thought to play a role in *Leishmania* promastigote growth and parasite virulence via interactions with host proteins, though its exact function is unclear [78, 79]. Likewise, adenylate cyclase is a suspected regulator of cAMP signaling during *Leishmania* differentiation [80, 81], but the exactly signaling pathways through which this is accomplished are unknown. Fatty acids have many potential roles within the cell related to membrane structure and composition, signaling, and energy. Fatty acid elongase family members are involved in fatty acid synthesis and may be involved in GPI anchoring [82], while sphingolipid delta 4 desaturase is involved in the degradation of sphingolipids, a process that has been linked to enabling *Leishmania* to survive the acidic environment of the phagolysosome once it is taken into host cells [83]. Genes that influence microtubule length and dynamics were systematically downregulated, including those that encode paraflagellar rod proteins, calmodulin-related proteins [84], multiple kinesins [85, 86], NIMA-related kinases [87], and mitogen-activated protein (MAP) kinase kinases [88–90]. These genes have been implicated in the regulation of flagellum length and mitotic spindle formation and may reflect changes in morphology and cell division that take place as the flagellated promastigote stage parasites transform into the aflagellated amastigotes. Consistent with previous microarray-based studies [27, 28], we also detected the downregulation of the known metacyclic markers SHERP and HASP as the parasite transformed from promastigotes to amastigotes.

Of the 14 GO categories enriched during the transition from 4 to 24-h amastigotes, those related to microtubules and protein complexes were specific to upregulated genes with many copies of β-tubulin, the primary component of microtubules, almost exclusively driving the GO analysis results. The upregulation of β-tubulin in early amastigotes compared to metacyclic promastigotes is noteworthy considering its downregulation in metacyclic promastigotes compared to procyclic promastigotes [55, 91]. Besides β-tubulin, many other top upregulated genes encoded surface antigens, including the developmentally-regulated amastigote-enriched protein amastin [27, 35, 44, 92]. GO categories related to glucose metabolism and oxidation-reduction processes were enriched among downregulated genes with many of the downregulated genes contributing to these categories belonging to the glycolysis pathway (GAPDH, pyruvate kinase, ENOL, triosephosphate isomerase, ALD, and PGI) or related to cholesterol/sterol metabolism (NSDHL, HMGR, FPPS, CYP51) or purine metabolism (XRPT). This may indicate a change in the metabolic requirements or preferences of *Leishmania* once the parasites have survived their initial entry into the mammalian cell. Other highly downregulated genes included heat shock proteins, biopterin transporters, and *meta1* [55, 74, 75].

A very significant dataset resulting from this study includes the large proportion of the DE genes which have no known function and are annotated as hypothetical proteins. Those make up 58 % (1724 of 2962) of the DE genes in the metacyclic to 4-h amastigote transition and 49 % (147 of 301) of the DE genes in the 4- to 24-h amastigote transition. While hypothetical genes have largely been overlooked to date, they almost certainly constitute an integral part of the transcriptomic signature of the parasites as members of co-expressed gene networks which are involved in common functional pathways or regulated by shared regulatory mechanisms.

## Conclusions

In this study, we performed transcriptomic profiling to identify genes that were differentially expressed in both parasite and host cells as *L. major* entered and persisted within murine macrophages during the first 72 h of an infection. The generation of RNA-seq data followed by the unambiguous mapping of reads from infected samples to the genomes of both the host and the parasite enabled us to identify genes that changed over the course of infection in the real-time context of a dynamic dual biological system, and to a much greater depth and sensitivity than has been previously reported. Collection of data from multiple biological replicates, the use of matched host control samples, careful statistical analysis of variation, and removal of batch effects provided us with a unique ability to detect biological differences between samples and timepoints with high confidence and sensitivity.

In addition to providing robust sets of markers for multiple developmental stages of *L. major* parasites and *Leishmania*-infected macrophages over several timepoints, this work contributes to a growing body of literature on the broader field of host-pathogen interactions. Indeed, the comprehensive dataset generated in this study will also serve as a reference for future studies using different *Leishmania* strains (or even different pathogens altogether) to examine infection of macrophages from multiple sources and in various states of activation, polarization, or rest. In conjunction with datasets that will be produced for other pathogens, a clearer picture of the signature of intracellular infection will emerge, providing additional insights into how pathogens are able to evade host defenses and modulate the biological functions of the cell in order to survive in the mammalian environment.

## Methods

### *Leishmania* culture

*Leishmania major* (clone V1, MHOM/IL/80/Friedlin) was isolated after passage through Balb/c mice. Promastigotes were grown in 50 % M199, 39 % Schneider’s medium along with 10 % FBS and 100 U/mL of Penicillin-Streptomycin at 25 **°**C. *L. major* promastigotes were not split for more than 5 passages to maintain virulence of the cultures. Enrichment for metacyclic promastigotes from stationary phase cultures was done by Ficoll density gradient centrifugation [93].

### Mouse infection

All procedures involving animals were approved by the Institutional Animal Care and Use Committee (IACUC) of the University of Maryland. Peritoneal macrophages were isolated from C57BL/6 mice (7 weeks, female) obtained from National Cancer Institute Charles River Laboratories by flushing the peritoneal cavity using 10 mL cold DPBS without calcium and magnesium. Macrophages isolated from ten mice were pooled for each experiment. Cells were plated in six well plates in 2.5 mL DMEM/F12 media supplemented with 10 % FBS, 1 % penicillin/streptomycin, and 1 % glutamine to a density of ~2.5 x 10^6^ cells/well and incubated overnight. Two hours after washing, cells were infected with Ficoll-enriched *L. major* metacyclic promastigotes at a ratio of 5 parasites per macrophage along with 5 % C5-deficient serum collected from DBA2 mice. Cells were lysed using the Trizol® reagent (Invitrogen, CA) at 4, 24, 48, and 72 h following infection.

### RNA isolation and cDNA library preparation

Total RNA was isolated using the Trizol® reagent (Invitrogen, CA), treated with DNase, and purified using the Qiagen RNeasy mini kit. RNA integrity was assessed using an Agilent 2100 bioanalyzer. Poly (A)-enriched cDNA libraries were generated using the Illumina TruSeq Sample Preparation kit (San Diego, CA) and checked for quality and quantity using the bioanalyzer and qPCR (KAPA Biosystems).

### RNA-seq data generation, pre-processing, and quality trimming

Paired end reads (100 bp) were obtained using the Illumina HiSeq 1500 platform. Trimmomatic [94] was used to remove any remaining Illumina adapter sequences from reads and to trim bases off the start or the end of a read when the quality score fell below a threshold of 20. Sequence quality metrics were assessed using FastQC [http://www.bioinformatics.babraham.ac.uk/projects/fastqc/].

### Mapping cDNA fragments to the reference genome, abundance estimation, and data normalization

Reads were aligned independently to the *L. major* genome (v. 6.0) obtained from the TriTrypDB database (www.tritrypdb.org) and to the mouse genome (v. mm10) from the UCSC genome browser (http://genome.ucsc.edu) using TopHat (v 2.0.10) [95]. Gene model annotations were provided for the mapping (option –G) with limitations on the identification of novel splice junctions (option –no-novel-juncs). Two mismatches per read were allowed (default parameter) and reads were allowed to map only to a single locus (option –g 1). The abundance of reads mapping to each gene feature in the aligned genome was determined using HTSeq[96]. Each resulting count table was restricted to protein-coding genes (23,100 genes for mouse and 8486 genes for *L. major*).

### Data quality assessment by statistical sample clustering and visualization

Multiple approaches were used to evaluate replicates and to visualize sample-sample distances. Those included Pearson correlation, median pairwise correlation analysis, box plots, Principal Component Analysis (PCA) and Euclidean distances-based hierarchical clustering. All components of the statistical pipeline, which we named cbcbSEQ, were done in R and can be accessed on GitHub (https://github.com/kokrah/cbcbSEQ/).

### Differential expression analysis

Non-expressed and weakly expressed genes, defined as having less than 1 read per million in n of the samples, where n is the size of the smallest group of replicates [97] (here *n* = 3), were removed prior to differential expression analysis. This is implemented by the filterCounts function within the cbcbSEQ R package (see above). A quantile normalization scheme was applied to all samples [98]. Following log2 transformation of the data, limma (a Bioconductor package) was used to conduct differential expression analyses. limma utilizes a standard variance moderated across all genes using a Bayesian model and produces *P* values with greater degrees of freedom [99]. The voom module was used to transform the data based on observational level weights derived from the mean-variance relationship prior to statistical modeling [100] (Additional file 2: Figure S6). Experimental batch effects were adjusted for by including experimental batch as a covariate in the statistical model [56]. Differentially expressed (DE) genes were defined as genes with a Benjamini-Hochberg multiple-testing adjusted *P* value of < 0.05.

### KEGG pathway analysis

KEGG pathway analysis using ConsensusPathDB-mouse was done to identify signaling and metabolic pathways that were over-represented in the mouse DE gene lists. For each KEGG pathway, a *P* value was calculated using a hypergeometric test and a cutoff of 0.01 was applied to identify enriched KEGG pathways. Genes that were DE more than 2-fold in *L. major*-infected cells relative to uninfected controls at each timepoint were used as input with up- and downregulated genes considered separately.

### Gene ontology (GO) analysis

GO categories enriched in the *L. major* DE gene lists were identified using the GOseq package in R [101]. GOseq was developed specifically to account for transcript length bias in GO analyses using RNA-seq data. For each comparison, upregulated and downregulated gene sets (no fold change cut-off) were input separately into GOseq. A *P* value cut-off of 0.05 was used.

### Availability of supporting data

The data sets supporting the results of this article are available in the NCBI Sequence Read Archive (SRA) repository [SRA: SRR1460724-SRR1460747, SRR1460767, SRR1460772, SRR2136702].

## Additional files

Additional file 1:
**Experimental design.** (XLSX 14 kb) Additional file 2: Supplementary figures and tables.
**Figure S1.** Global assessment of data distribution. **Figure S2.** Principal Component Analysis (PCA) and hierarchical clustering analysis prior to accounting for batch effects. **Figure S3.** Global changes in murine macrophage gene expression upon infection by *L. major*. **Figure S4.** Expression patterns for the top 20 up- and down-regulated genes in *L. major*-infected murine macrophages at 4, 24, 48, and 72 hpi. **Figure S5.** Global changes in *L. major* and murine macrophage gene expression over the course of infection. **Figure S6.** Mean-variance curve modeling and fitting of a local regression trend line by voom. **Table S1.** KEGG pathways enriched among genes upregulated in *L. major*-infected murine macrophages at 24, 48, and 72 hpi. **Table S2.** KEGG pathways enriched among genes downregulated in *L. major*-infected murine macrophages at 24, 48, and 72 hpi. (PDF 25627 kb) Additional file 3:
**Differentially expressed gene lists for uninfected vs.** ***L. major*** **-infected murine macrophages within and across time using limma.** (XLS 2631 kb) Additional file 4:
**Enriched KEGG pathways and corresponding murine differentially expressed genes.** (XLS 228 kb) Additional file 5:
**Comparison of differentially expressed murine gene lists with published literature.** (XLS 267 kb) Additional file 6:
**Differentially expressed gene lists for** ***L. major*** **parasite stages and timepoints using limma.** (XLS 547 kb) Additional file 7:
**Enriched GO categories and corresponding L. major differentially expressed genes.** (XLS 178 kb)

## Abbreviations

amast: amastigotes
BP: biological process
CC: cellular component
DE: differentially expressed
FC: fold change
GO: gene ontology
hpi: hours post-infection
inf: infected
metac: metacyclic promastigotes
MF: molecular function
PC: principal component
PCA: principal component analysis
SRA: Sequence Read Archive
Th: T helper
uninf: uninfected
