# Supplementary material for: Simultaneous transcriptional profiling of Leishmania major and its murine macrophage host cell reveals insights into host-pathogen interactions
Source: BMC Genomics. 2015 Dec 29;16:1108. doi: 10.1186/s12864-015-2237-2 (PMC4696162; doi:10.1186/s12864-015-2237-2)
Supplement: Additional file 2: Supplementary figures and tables. — Figure S1. Global assessment of data distribution. Figure S2. Principal Component Analysis (PCA) and hierarchical clustering analysis prior to accounting for batch effects. Figure S3. Global changes in murine macrophage gene expression upon infection by L. major. Figure S4. Expression patterns for the top 20 up- and down-regulated genes in L. major-infected murine macrophages at 4, 24, 48, and 72 hpi. Figure S5. Global changes in L. major and murine macrophage gene expression over the course of infection. Figure S6. Mean-variance curve modeling and fitting of a local regression trend line by voom. Table S1. KEGG pathways enriched among genes upregulated in L. major-infected murine macrophages at 24, 48, and 72 hpi. Table S2. KEGG pathways enriched among genes downregulated in L. major-infected murine macrophages at 24, 48, and 72 hpi. (PDF 25627 kb) [file 12864_2015_2237_MOESM2_ESM.pdf]

Additional File 2: Supplementary Figures and Tables

This file contains:

Figures S1 to S6

Tables S1 and S2

Supplementary Figure Legends

TABLE OF CONTENTS

Figure S1 Global assessment of data distribution.

Figure S2 Principal Component Analysis (PCA) and hierarchical clustering analysis prior to accounting for batch effects.

Figure S3 Global changes in murine macrophage gene expression upon infection by *L. major*.

Figure S4 Expression patterns for the top 20 up- and down-regulated genes in *L. major*-infected murine macrophages at 4, 24, 48, and 72 hpi.

Figure S5 Global changes in *L. major* and murine macrophage gene expression over the course of infection.

Figure S6 Mean-variance curve modeling and fitting of a local regression trend line by `voom`.

Table S1 KEGG pathways enriched among genes upregulated in *L. major*-infected murine macrophages at 24, 48, and 72 hpi.

Table S2 KEGG pathways enriched among genes downregulated in *L. major*-infected murine macrophages at 24, 48, and 72 hpi.

Figure S1

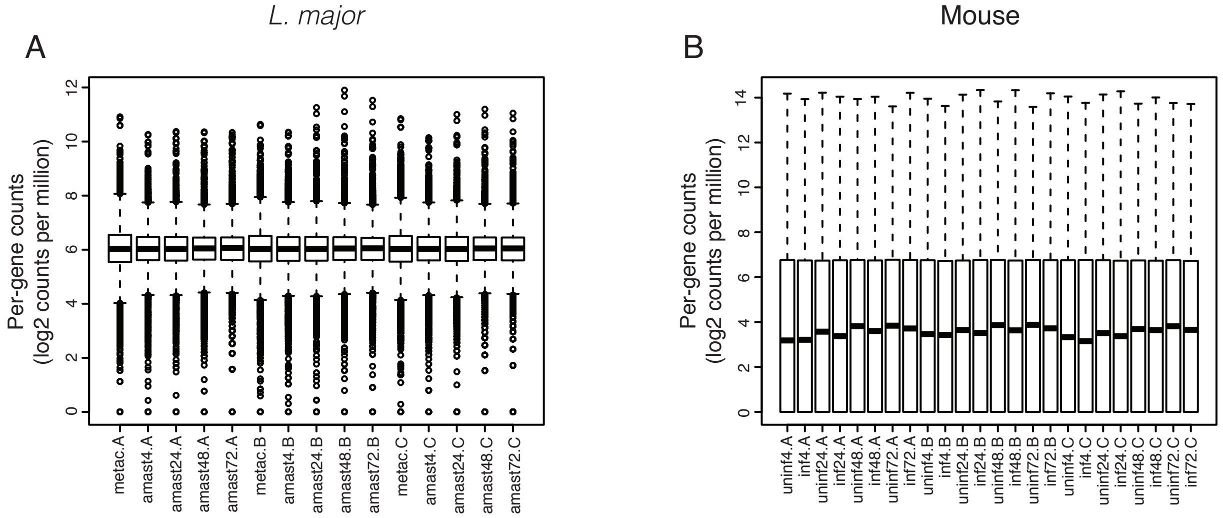

L. major
Mouse
A
B
Per-gene counts
(log2 counts per million)
metac.A
amast4.A
amast24.A
amast48.A
amast72.A
metac.B
amast4.B
amast24.B
amast48.B
amast72.B
metac.C
amast4.C
amast24.C
amast48.C
amast72.C
uninf4.A
inf4.A
uninf24.A
inf24.A
uninf48.A
inf48.A
uninf72.A
inf72.A
uninf4.B
inf4.B
uninf24.B
inf24.B
uninf48.B
inf48.B
uninf72.B
inf72.B
uninf4.C
inf4.C
uninf24.C
inf24.C
uninf48.C
inf48.C
uninf72.C
inf72.C

Figure S2

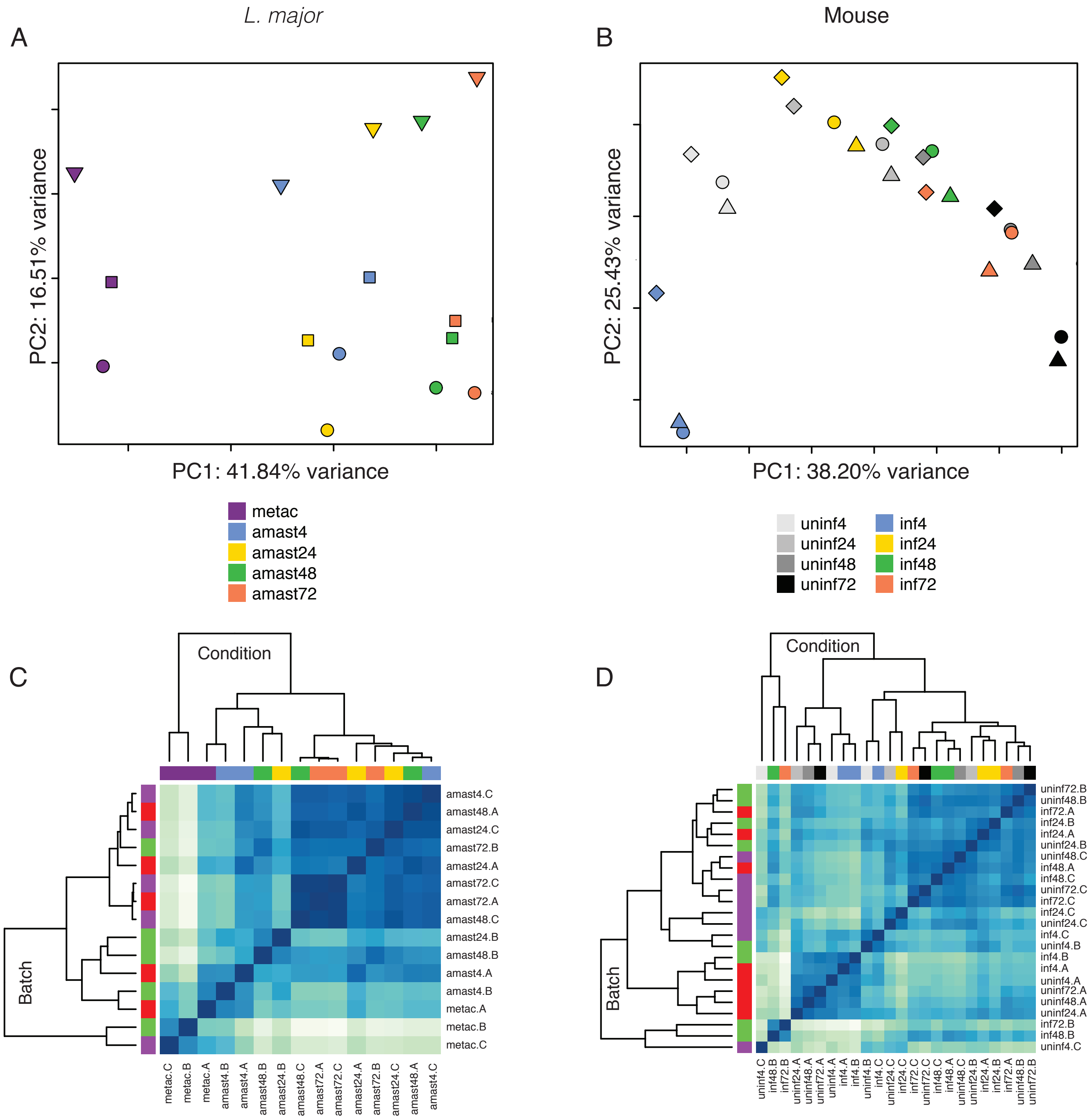

L. major
A
PC2: 16.51% variance
PC1: 41.84% variance
metac
amast4
amast24
amast48
amast72
Mouse
B
PC2: 25.43% variance
PC1: 38.20% variance
uninf4
uninf24
uninf48
uninf72
inf4
inf24
inf48
inf72
C
Condition
Batch
amast4.C
amast48.A
amast24.C
amast72.B
amast24.A
amast72.C
amast72.A
amast48.C
amast24.B
amast48.B
amast4.A
amast4.B
metac.A
metac.B
metac.C
metac.C
metac.B
metac.A
amast4.B
amast4.A
amast48.B
amast24.B
amast48.C
amast72.A
amast72.C
amast24.A
amast72.B
amast24.C
amast48.A
amast4.C
D
Condition
Batch
uninf72.B
uninf48.B
inf72.A
inf24.B
inf24.A
uninf24.B
uninf48.C
inf48.A
inf48.C
uninf72.C
inf72.C
inf24.C
uninf24.C
inf4.C
uninf4.B
inf4.B
inf4.A
uninf4.A
uninf72.A
uninf48.A
uninf24.A
inf72.B
inf48.B
uninf4.C
uninf4.C
inf48.B
inf72.B
uninf24.A
uninf48.A
uninf72.A
uninf4.A
inf4.A
inf4.B
uninf4.B
inf4.C
uninf24.C
inf24.C
inf72.C
uninf72.C
inf48.C
inf48.A
uninf48.C
uninf24.B
inf24.A
inf24.B
inf72.A
uninf48.B
uninf72.B

Figure S3

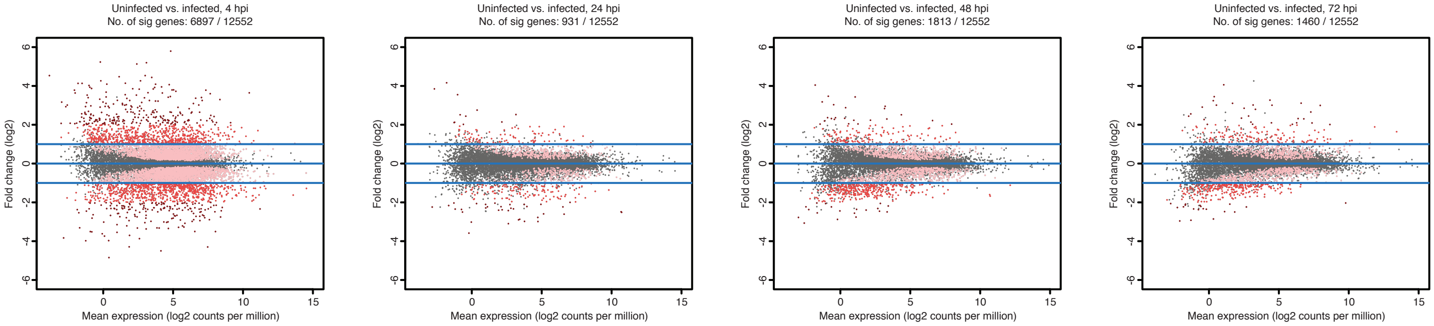

Uninfected vs. infected, 4 hpi
No. of sig genes: 6897 / 12552
Uninfected vs. infected, 24 hpi
No. of sig genes: 931 / 12552
Uninfected vs. infected, 48 hpi
No. of sig genes: 1813 / 12552
Uninfected vs. infected, 72 hpi
No. of sig genes: 1460 / 12552
Fold change (log2)
Mean expression (log2 counts per million)

Figure S4

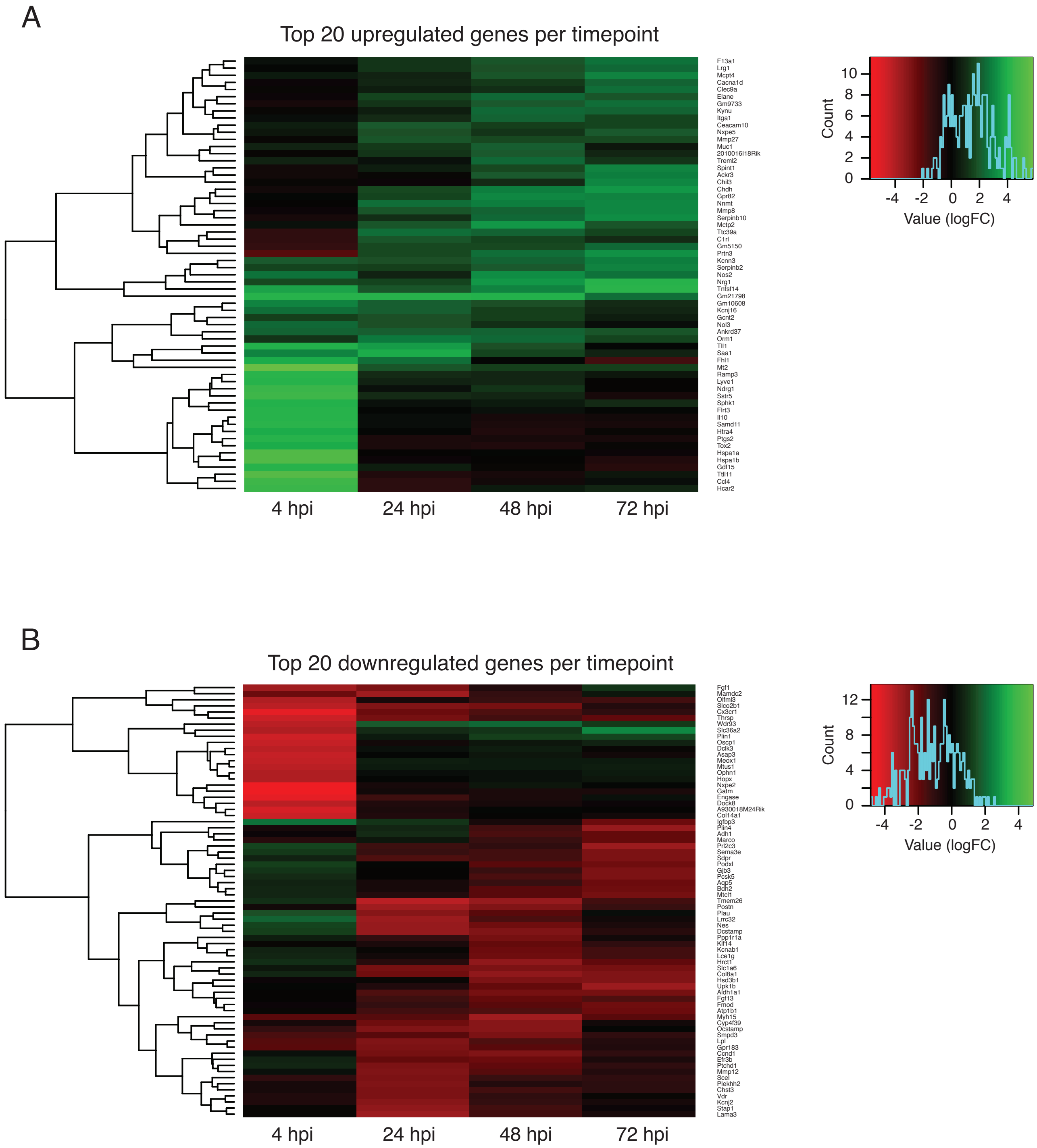
A
Top 20 upregulated genes per timepoint
F13a1
Lrg1
Mcpt4
Cacna1d
Clec9a
Elane
Gm9733
Kynu
Itga1
Ceacam10
Nxpe5
Mmp27
Muc1
2010016I18Rik
Treml2
Spint1
Ackr3
Chil3
Chdh
Gpr82
Nnmt
Mmp8
Serpinb10
Mctp2
Ttc39a
C1rl
Gm5150
Prtn3
Kcnn3
Serpinb2
Nos2
Nrg1
Tnfsf14
Gm21798
Gm10608
Kcnj16
Gcnt2
Nol3
Ankrd37
Orm1
Tll1
Saa1
Fhl1
Mt2
Ramp3
Lyve1
Ndrg1
Sstr5
Sphk1
Flrt3
Il10
Samd11
Htra4
Ptgs2
Tox2
Hspa1a
Hspa1b
Gdf15
Ttll11
Ccl4
Hcar2
4 hpi
24 hpi
48 hpi
72 hpi
Count
10
8
6
4
2
0
-4
-2
0
2
4
Value (logFC)
B
Top 20 downregulated genes per timepoint
Fgf1
Mamdc2
Olfml3
Slco2b1
Cx3cr1
Thrsp
Wdr93
Slc36a2
Plin1
Oscp1
Dclk3
Asap3
Meox1
Mtus1
Ophn1
Hopx
Nxpe2
Gatm
Engase
Dock8
A930018M24Rik
Col14a1
Igfbp3
Plin4
Adh1
Marco
Prl2c3
Sema3e
Sdpr
Podxl
Gjb3
Pcsk5
Aqp5
Bdh2
Mtcl1
Tmem26
Postn
Plau
Lrrc32
Nes
Dcstamp
Ppp1r1a
Kif14
Kcnab1
Lce1g
Hrct1
Slc1a6
Col8a1
Hsd3b1
Upk1b
Aldh1a1
Fgf13
Fmod
Atp1b1
Myh15
Cyp4f39
Ocstamp
Smpd3
Lpl
Gpr183
Ccnd1
Efr3b
Ptchd1
Mmp12
Scel
Plekhh2
Chst3
Vdr
Kcnj2
Stap1
Lama3
4 hpi
24 hpi
48 hpi
72 hpi
Count
12
8
4
0
-4
-2
0
2
4
Value (logFC)

Figure S5

A

Leishmania major

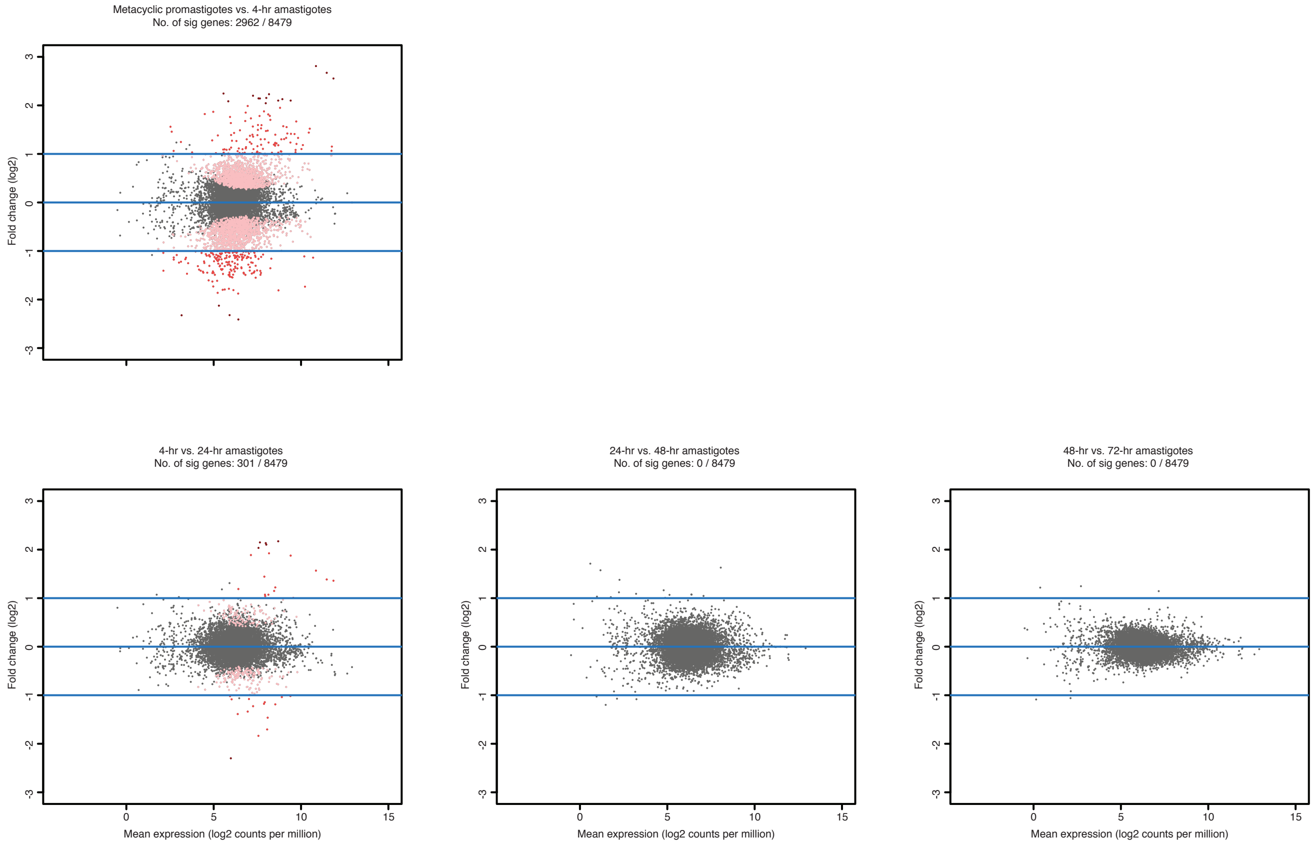

B

Mouse peritoneal macrophages

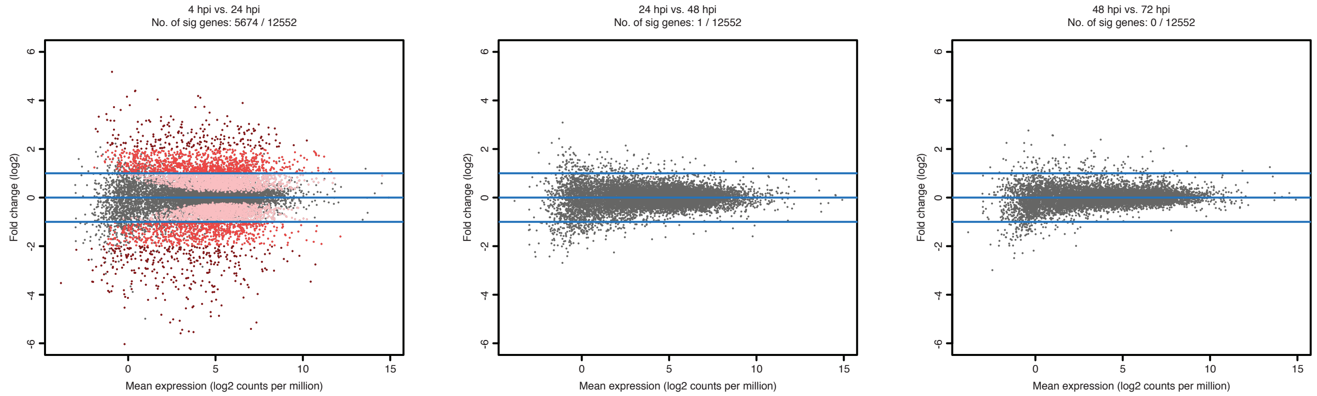

Figure S6

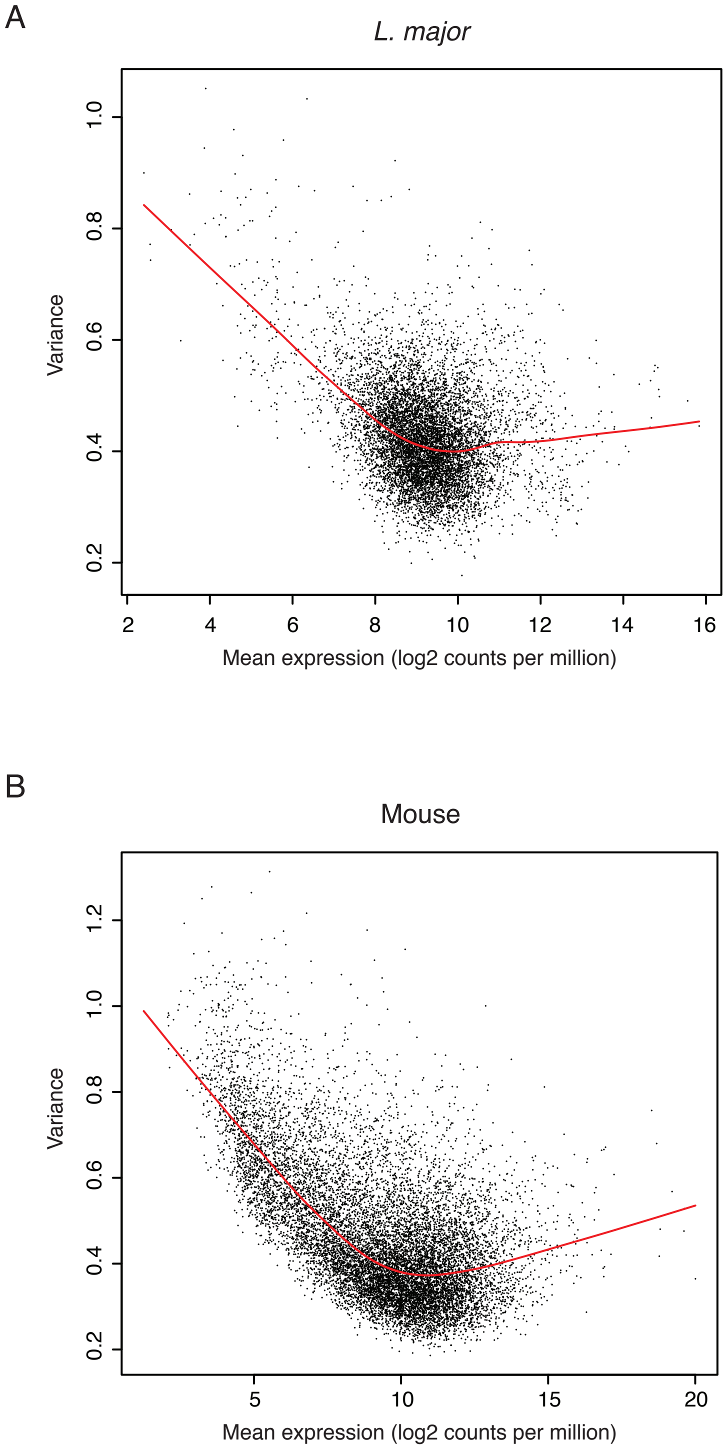
A
L. major
Variance
0.2
0.4
0.6
0.8
1.0
2
4
6
8
10
12
14
16
Mean expression (log2 counts per million)
B
Mouse
Variance
0.2
0.4
0.6
0.8
1.0
1.2
5
10
15
20
Mean expression (log2 counts per million)

Table S1. KEGG pathways enriched among genes upregulated in *L. major*-infected murine macrophages at 24, 48, and 72 hpi.

**KEGG pathway upregulated, 24 hpi**	**Number DE Genes**	**Pathway Size**	***P* Value**
Glycolysis / Gluconeogenesis	4	65	2.88e-06
Chemical carcinogenesis	6	95	2.62e-05
Metabolism of xenobiotics by cytochrome P450	6	96	2.78e-05
Drug metabolism - cytochrome P450	6	97	2.95e-05
Starch and sucrose metabolism	4	54	3.44e-04
Glutathione metabolism	4	55	3.70e-04
Staphylococcus aureus infection	3	52	4.11e-03
HIF-1 signaling pathway	2	113	5.33e-03
Natural killer cell mediated cytotoxicity	2	119	6.40e-03
Osteoclast differentiation	4	126	7.80e-03

**KEGG pathway upregulated, 48 hpi**	**Number DE Genes**	**Pathway Size**	***P* Value**
Osteoclast differentiation	8	126	9.58e-07

**KEGG pathway upregulated, 72 hpi**	**Number DE Genes**	**Pathway Size**	***P* Value**
Osteoclast differentiation	10	126	4.94e-08
Staphylococcus aureus infection	5	52	1.68e-04
Calcium signaling pathway	8	183	5.06e-04
Chemokine signaling pathway	8	199	8.78e-04
Hematopoietic cell lineage	5	87	1.81e-03
Nicotinate and nicotinamide metabolism	3	32	3.99e-03
Leishmaniasis	4	66	4.34e-03
Amoebiasis	5	120	7.19e-03
Salivary secretion	4	77	7.49e-03
Complement and coagulation cascades	4	77	7.49e-03
Tuberculosis	6	177	8.87e-03

Table S2. KEGG pathways enriched among genes downregulated in *L. major*-infected murine macrophages at 24, 48, and 72 hpi.

KEGG pathway downregulated, 24 hpi	Number DE Genes	Pathway Size	*P* Value
Transcriptional misregulation in cancer	10	180	2.10e-05
Pathways in cancer	13	326	4.09e-05
Focal adhesion	10	206	6.63e-05
Melanoma	6	73	1.21e-04
PI3K-Akt signaling pathway	12	353	3.64e-04
Proteoglycans in cancer	9	228	7.14e-04
Glycosaminoglycan biosynthesis - chondroitin sulfate/dermatan sulfate	3	20	1.17e-03
Small cell lung cancer	5	86	2.20e-03
Thyroid cancer	3	29	3.51e-035
Wnt signaling pathway	6	143	4.18e-03
Colorectal cancer	4	64	4.74e-03
Hippo signaling pathway	6	156	6.36e-03
Amoebiasis	5	120	9.04e-03

KEGG pathway downregulated, 48 hpi	Number DE Genes	Pathway Size	*P* Value
Cell cycle	20	128	7.91e-14
Progesterone-mediated oocyte maturation	9	88	4.20e-06
p53 signaling pathway	8	69	2.25e-05
Oocyte meiosis	10	112	4.28e-05
ECM-receptor interaction	8	87	7.47e-04
Focal adhesion	13	206	7.82e-04
Pathways in cancer	17	326	1.13e-03
Hippo signaling pathway	10	156	2.78e-03
Small cell lung cancer	7	86	3.21e-03
Homologous recombination	4	28	3.37e-03
HTLV-I infection	13	279	4.30e-03
Amoebiasis	8	120	5.71e-03
Fanconi anemia pathway	5	52	6.12e-03

KEGG pathway downregulated, 72 hpi	Number DE Genes	Pathway Size	*P* Value
ECM-receptor interaction	15	87	1.08e-10
Focal adhesion	20	206	3.31e-09
PI3K-Akt signaling pathway	21	353	5.47e-06
Pathways in cancer	20	326	5.86e-06
Protein digestion and absorption	10	88	7.90e-06
p53 signaling pathway	7	69	5.63e-05
Cell cycle	9	128	2.02e-04
Small cell lung cancer	8	86	2.69e-04
Amoebiasis	9	120	5.66e-04
Oocyte meiosis	8	112	1.57e-03
Progesterone-mediated oocyte maturation	6	88	1.65e-03
Hippo signaling pathway	9	156	3.53e-03
Tyrosine metabolism	4	43	9.68e-03

SUPPLEMENTARY FIGURE LEGENDS

Figure S1. Global assessment of data distribution. For each sample, the counts of reads mapping to *L. major* (**A**) and mouse (**B**) were normalized for sequencing library size and a box plot was generated to compare the distribution of per-gene counts (log2 counts per million with an offset of 1). The ends of the whiskers represent the lowest datum still within 1.5 interquartile range (IQR) of the lower quartile, and the highest datum still within 1.5 IQR of the upper quartile. Gene features with extremely high or low expression levels are shown as open circles above and below the whiskers, respectively. Samples are named according to sample type ("metac" for *L. major* metacyclic promastigotes, "amast" for *L. major* amastigotes, "uninf" for uninfected mouse macrophage, or "inf" for *L. major*-infected mouse macrophage), timepoint (4, 24, 48, or 72 hpi) and experimental batch (A, B, or C) (see **Additional file 1**).

Figure S2. Principal Component Analysis (PCA) and hierarchical clustering analysis prior to accounting for batch effects. RNA-seq was carried out on mouse macrophages infected with *L. major* at 4, 24, 48, and 72 hpi as well as on the metacyclic promastigotes used for the infection. Principal component analysis (PCA) plots and heatmaps of hierarchical clustering analyses using Euclidean distance are shown for the *L. major* (**A, C**) and mouse (**B, D**) transcriptomes over the course of the experiment. The analyses were performed using all annotated protein-coding genes following filtering for low counts and quantile normalization prior to accounting for batch effects in the statistical model (8,479 genes for *L. major* and 12,552 genes for mouse). In the PCA plots, the first two principal components are shown on the X and Y axes, respectively, with the proportion of total variance attributable to that PC indicated. Each experimental sample is represented as a single point with color indicating sample type/timepoint and shape indicating experimental batch. Colors along the tops of the heatmaps indicate the sample type/timepoint and colors along the left sides of the heatmaps indicate the experimental batch. Samples are named according to sample type ("metac" for *L. major* metacyclic promastigotes, "amast" for *L. major* amastigotes, "uninf" for uninfected mouse macrophage, or "inf" for *L. major*-infected mouse macrophage), timepoint (4, 24, 48, or 72 hpi) and experimental batch (A, B, or C) (see **Additional file 1**).

Figure S3. Global changes in murine macrophage gene expression upon infection by *L. major*. Differential expression analysis was done to compare infected mouse macrophages and uninfected controls at 4, 24, 48, and 72 hpi using `limma` after `voom` transformation, taking experimental batch into account as part of the `limma` statistical model. The MA plots show the

relationship between mean expression (log2 counts per million with an offset of 0.5) and fold change (log2) for each timepoint. Each point represents one gene. Genes upregulated in infected samples relative to uninfected samples exhibit positive fold changes, while downregulated genes exhibit negative fold changes. Points colored in gray represent genes that were not significantly different between uninfected and infected macrophages (*P* value < 0.05) while points colored in shades of red represent significant genes, with those showing a < 2-fold difference (logFC < 1) colored in pink, between 2- and 4-fold difference colored in red, and > 4-fold difference colored in burgundy.

Figure S4. Expression patterns for the top 20 up- and down-regulated genes in *L. major*-infected murine macrophages at 4, 24, 48, and 72 hpi. A heatmap is used to illustrate the pattern of changes in gene expression over time for the top differentially expressed genes in uninfected vs. *L. major*-infected murine macrophages. The top 20 significantly up-regulated (**A**) and down-regulated (**B**) genes at each timepoint (*P* value < 0.05) were selected for inclusion. A color key and histogram for the frequency of fold change values is shown with each panel.

Figure S5. Global changes in *L. major* and murine macrophage gene expression over the course of infection. Differential expression analysis was done to compare changes in *L. major* metacyclic promastigotes and intracellular amastigote gene expression (**A**) and in *L. major*-infected mouse macrophages relative to uninfected controls (**B**) over the time course of the experiment (4, 24, 48, and 72 hpi). Comparisons were done using `limma` after `voom` transformation, taking experimental batch into account as part of the `limma` statistical model. The MA plots show the relationship between mean expression (log2 counts per million with an offset of 0.5) and fold change (log2). Each point represents one gene. Genes upregulated in the second of the specified timepoints relative to the first are shown as exhibiting positive fold changes, while downregulated genes exhibit negative fold changes. Points colored in gray represent genes that were not significantly different between the tested conditions (*P* value < 0.05) while points colored in shades of red represent significant genes, with those showing a < 2-fold difference (logFC < 1) colored in pink, between 2- and 4-fold difference colored in red, and > 4-fold difference colored in burgundy.

Figure S6. Mean-variance curve modeling and fitting of a local regression trend line by `voom`. After log-transformation of the quantile-normalized data, the `voom` function in `limma` was used to compute the mean-variance relationship and to generate gene weights for use in the subsequent differential expression analysis. The relationships between mean expression (log2 counts per

million with an offset of 0.5) and variance were modeled by `voom` for *L. major* (**A)** and mouse (**B**). For each, a trend line was created using a local regression (loess). Trend line values (red line) are robust to genes with high variability and were used as gene weights by `limma`.

Table S1. KEGG pathways enriched among genes upregulated in *L. major*-infected murine macrophages at 24, 48, and 72 hpi. KEGG pathway analysis using ConsensusPathDB identified signaling and metabolic pathways that were over-represented among genes upregulated > 2-fold in *L. major*-infected mouse macrophages at 24, 48, and 72 hpi (*P* value < 0.01) relative to uninfected controls. For each enriched KEGG pathway, the number of differentially expressed (DE) genes belonging to that pathway, the total number of genes in the pathway, and the *P* value for the enrichment are reported. The DE genes corresponding to each enriched KEGG pathway are reported in **Additional file 4**.

Table S2. KEGG pathways enriched among genes downregulated in *L. major*-infected murine macrophages at 24, 48, and 72 hpi. KEGG pathway analysis using ConsensusPathDB identified signaling and metabolic pathways that were over-represented among genes downregulated > 2-fold in *L. major*-infected mouse macrophages at 24, 48, and 72 hpi (*P* value < 0.01) relative to uninfected controls. For each enriched KEGG pathway, the number of differentially expressed (DE) genes belonging to that pathway, the total number of genes in the pathway, and the *P* value for the enrichment are reported. The DE genes corresponding to each enriched KEGG pathway are reported in **Additional file 4**.
